# Retrospective analysis of rapid drug desensitization with biologic agents: A single center experience

**DOI:** 10.1002/clt2.12397

**Published:** 2024-10-21

**Authors:** Döne Gülçin Unutmaz Erkaya, Makbule Seda Bayrak Durmaz, Begüm Görgülü Akın, Sevim Bavbek

**Affiliations:** ^1^ Division of Immunology and Allergy Department of Chest Diseases Ankara University School of Medicine Ankara Turkey

**Keywords:** biologics, breakthrough reaction, hypersensitivity reactions, monoclonal antibodies, rapid drug desensitization

## Abstract

**Background:**

Following the increased use of biological agents, a subset of patients experiences hypersensitivity reaction (HSR). We reported our experience with rapid drug desensitization (RDD) to nine biologics (rituximab, infliximab, cetuximab, trastuzumab, pertuzumab, nivolumab, brentuximab, tocilizumab and filgrastim) and identified risk factors for breakthrough reactions (BTRs).

**Method:**

This was a retrospective review (2013–2022) of patients with immediate HSRs to biological agents. Initial HSRs were classified as grade 1, 2, or 3 in their severity. Skin prick tests (SPT)/intradermal tests (IDT) were performed using implicated agents. The phenotypes of HSRs were defined as Type I, cytokine‐release syndrome (CRS), mixed reactions (cytokine‐release + type I) based on history, clinical presentations and skin tests with implicated biologicals. A 12‐step RDD protocol was used.

**Results:**

The study comprised 45 patients (F/M: 31/14, median age: 55 (range: 20–69)). Majority of the patients reacted at the first infusion (*n*: 29/45, 64.4%). The majority of initial HSRs were grade 3 (*n*: 24/45, 53.3%) and grade 2 (*n*: 21/45, 46.6%); none were grade 1. Initial reactions were presented as type I (*n*: 20/45, 44.4%), CRS (*n*: 12/45, 26.6%) and mixed (*n*: 13/45, 28.8%). A total of 258 RDDs were performed and 98.4% of them were completed successfully. BTRs occurred in 36/258 (13.9%) infusions of RDDs. There was no significant association between the BTRs and age, drug cycle, SPT and IDT positivity, gender, comorbidities, or atopy.

**Conclusion:**

In our experience, 98.4% of 258 RDDs to biologics were successfully completed; RDD was safe and effective for our population.

## 1 INTRODUCTION

Biologic agents treat a variety of conditions and they are the cornerstone of the targeted therapies for malignancies (solid tumors e.g. breast cancer, hematological malignancies e.g. Non‐Hodgkin lymphoma, Chronic lymphocytic leukemia), inflammatory bowel disease (Crohn's disease, ulcerative colitis), autoimmune (rheumatoid arthritis, systemic lupus erythematosus), and allergic diseases (asthma, chronic idiopathic urticaria, atopic dermatitis).^1^, ^2^, ^3^, ^4^, ^5^ Following the increased use of biological agents, a subset of patient experiences adverse reactions including hypersensitivity reaction (HSR) that limits their use.^5^

The phenotypes of HSRs to biologics were originally defined as type I (IgE/non‐IgE), infusion‐related, cytokine‐release syndrome (CRS), mixed reactions (cytokine‐release + type I), type III, and type IV.^5^ In this paper, we aimed to study immediate HSRs, including type I, CRS, and mixed reactions. We did not include the patients with Type III and Type IV reactions since they are not considered as good candidates for rapid drug desensitization (RDD). In this paper we aimed to report our experience with RDD in nine biologics.

According to previous studies, the rate of immediate HSR is 5%–10% for rituximab,^6^ 2%–3% for infliximab,^7^, ^8^, ^9^ 0.6%–5% for trastuzumab.^3^, ^10^ The rate of severe reaction is 1.1%–5% for cetuximab.^11^ Hypersensitivity reactions have also been described for brentuximab, nivolumab, pertuzumab and tocilizumab.^5^

When a patient experiences an immediate HSR to a biological or any therapeutic agent, the most frequently chosen action is to stop the culprit drug even in responsive patients and switch to an alternative medication. If the culprit drug is the best medication or the only choice for the patient, the sustainability of appropriate treatment can be allowed by RDD. RDD is a useful choice for the administration of various parenteral chemotherapeutic agents, including biological agents that cause immediate type HSR by inducing temporary tolerance. Many desensitization protocols for desensitization to biologics have been developed by different groups, but the 12‐step RDD protocol developed at Brigham and Women's Hospital (BWH; Boston, MA, USA) is considered a well validated protocol based on several hundred cases.^12^, ^13^, ^14^, ^15^

We have been applying the RDD protocol developed at the BWH for patients with immediate‐type HSRs to biologics since 2013. The protocol provides the induction of temporary tolerance to biologics and suits the local features of our center.^14^, ^15^, ^16^

In this paper, we first aimed to report our experience with RDD with nine biologics, such as rituximab, infliximab, cetuximab, trastuzumab, pertuzumab, nivolumab, brentuximab, tocilizumab, and filgrastim. Our second purpose was to identify the risk factors for breakthrough reactions (BTRs) during RDD with biological agents. To our knowledge, this is the largest series of biological desensitizations reported in Turkey.

## 2 MATERIALS & METHODS

### 2.1 Study design

The study was designed as a retrospective chart review (2013–2022) of patients with symptoms of immediate HSRs to biological agents. Informed consent was obtained from all participants. We used the same desensitization protocol, 12‐step RDD protocol developed at Brigham and Women's Hospital (BWH; Boston, MA, USA) with chemotherapeutics and biologics as in our previous trial.^14^, ^15^, ^16^ The study was approved by the local Ethics Committee of the School of Medicine, Ankara University (Approval number: I11‐727‐21, 2022).

### 2.2 Subjects

Patients who presented to the Department of Chest Diseases and the Division of Clinical Immunology and Allergy, School of Medicine, Ankara University, with symptoms of immediate‐type HSRs to biological drugs were eligible for RDD. Reactions that occurred during or within 1 h of biological infusion were considered immediate‐type reactions. As in the BWH‐based protocol, immediate HSRs were classified as mild (grade I), moderate (grade II), or severe (grade III) in accordance with Brown's grading system. HSRs were recorded and classified as mild (grade 1, symptoms limited to skin), moderate (grade 2, Symptoms involve at least two organs/systems (e.g., flushing and dyspnea), but there is no signiflcant decrease in blood pressure or oxygen saturation), or severe (grade 3, Severe symptoms typically involve at least 2 organs/systems, and there is a signiflcant decrease in blood pressure (systolic <90 mm Hg and/or syncope) and/or oxygen saturation <92%) according to Brown's classification. A temperature higher than 38°C was also classified as a moderate reaction.^17^ This allowed us to compare our own data with original BWH‐derived reports. Signs and symptoms of HSRs were defined as cutaneous (flushing, pruritus, urticaria, or angioedema), cardiovascular (chest pain, tachycardia, sense of impending doom, presyncope, syncope, and hypotension), respiratory (naso‐ocular symptoms, dyspnea, wheezing and oxygen desaturation, and throat tightness), gastrointestinal (nausea, vomiting, diarrhea, and abdominal pain), and atypical manifestations (fever/chills, back and neck pain, and numbness/weakness).^17^

### 2.3 Classification of reactions

The phenotypes of HSRs to biologics were originally defined as type I (IgE/non‐IgE), infusion‐related, CRS, mixed reactions (cytokine‐release + type I), type III, and type IV.^5^, ^18^, ^19^

Phenotypes were defined based on clinical presentations and endotypes were defined based on skin testing and tryptase levels. Type I reactions (IgE or non–IgE‐mediated) were defined as flushing, pruritus, urticaria, shortness of breath, wheezing, hypotension, and life‐threatening anaphylaxis, which indicated massive release of histamine. Skin test positivity to biologics was considered an IgE‐mediated reaction. CRS was defined as fever/chills, nausea, pain, headache, and rigor not responding to premedication/slower infusion rate during the first infusion. A mixed reaction was defined as wheezing, flushing, urticaria, pruritus, and/or a combination of skin test positivity and increased tryptase levels with fever/chills, nausea, pain, headache, and rigor.^5^

### 2.4 Evaluation of atopy

Skin prick tests (SPT) were performed using a common inhalant allergen panel (Allergopharma, Stockholm, Sweden) as usual. Histamine (10 mg/mL) and phenolated glycerol saline were used as the positive and negative controls, respectively. A mean wheal diameter of ≥3 mm more than the negative control solution was considered positive.

### 2.5 Serum tryptase measurement

Serum tryptase measurement was performed from the blood samples obtained using the enzyme‐linked immunosorbent test radioimmunoassay (ImmunoCAP 100) at the basal level and as early as within 30 min during BTR. Basal tryptase was measured in all subjects as part of the 12‐step protocol. The basal level is normally considered less than 11.5 mg/L. A significant elevation in tryptase was considered based on the following equation: baseline tryptase multiplied by 1.2 plus 2 mg/L.^20^

### 2.6 Serum IL‐6 measurement

Serum Levels of IL‐6 were measured using a Beckman Coulter Access autoanalyzer and the chemiluminescent immunoassay method (Access Immunoassay System, Beckman Coulter).

### 2.7 Skin testing with biologics

Skin testing with implicated biologics was performed in all subjects at least 2–4 weeks after the initial reaction. We started the procedure with a SPT, using the undiluted form of the drug for each biological. If SPT was negative, intradermal tests (IDT) was performed using published nonirritating concentrations. IDT was considered positive if the initial edema increased by at least 3 mm in diameter and was surrounded by erythema after 20 min.^21^

### 2.8 Desensitization protocol

All desensitization protocols and premedication were performed in inpatient settings using the suggestion of BWH's RDD protocol. The desensitization protocol used was the 3‐bag (with 250 mL of 5% dextrose or saline) 12‐step desensitization protocol developed by BWH (Table S1). Briefly, 3‐bag with different concentrations (X/100 mg, X/10 mg, and X mg, respectively, X being the non‐diluted drug concentration) were infused for 6 h in 12 consecutive steps at increasing rates and concentrations (doubling doses) every 15 min until the final rate of 60–80 cc/h was reached. For some patients with severe initial HSR, the protocol was modified as a 4‐bag 16‐step protocol, in which the first solution was diluted at X/1000 mg.^22^, ^23^, ^24^, ^25^ Premedication was performed according to the patients' standard premedication protocol including glucocorticoids (methylprednisolone 16–40 mg intravenously), H1 (pheniramine 45.5 mg intravenously), and H2 antihistamine (famotidine 20 mg or ranitidine 50 mg intravenously). For patients with cutaneous reactions or bronchospasm during initial desensitization, the protocol was adjusted by adding aspirin and montelukast, respectively, to the premedication regimen, given the data regarding their benefit in patients with such symptoms.^26^ Patients were asked to pause their beta‐adrenergic blocking medication for 24 h before desensitization.^18^, ^19^, ^26^

As an experienced department in RDD, we provided one‐to‐one desensitization‐trained nursing care for each desensitization.^14^, ^15^, ^16^

For BTR during RDD, the infusion was paused, and medications were administered based on the type of symptom. After the reaction was resolved, the protocol was resumed for subsequent desensitization, and additional premedication was added before the step where the previous reaction occurred.

### 2.9 Statistical analysis

Statistical analyses were performed using IBM^®^ SPSS software version 28. Descriptive statistics were presented as frequency (percentage) or median (min‐max). The *χ* ^2^ and Exact tests were used to compare the proportions in different categorical groups. Paired nominal data were compared with McNemar's test. Continuous variables were investigated with visual and analytical methods to determine the normal distribution and analyzed with the Mann–Whitney *U* test or Kruskal Wallis test where appropriate. The Wilcoxon signed rank test was used to compare two non‐parametric related samples. An overall type‐1 error level was used to infer statistical significance.

## 3 RESULTS

The study group consisted of 31 women and 14 men. The median age was 55 years (range: 20–69). Forty‐five patients had 258 RDDs with nine different biologics. The demographic and clinical characteristics of the study group and implicated biological agents are detailed in Tables 1 and 2.

**TABLE 1:** Demographics and clinical characteristics of the study group.

	Patients, *n* (%)
Age (years; median (range))	55 (20–69)
Sex	
Female	31 (68.9)
Male	14 (31.1)
Atopy^a^	
Atopic	5/21 (23.8)
Pollen	3 (14.3)
Mite	2 (9.5)
Non‐atopic	16/21 (76.2)

	Tested patients, *n* (%)	Positivity ST result, *n* (%)	Positive test concentration
Skin test with culprit biologics	40/45 (88.8)	10/40 (25)	
Biologic agent			
Rituximab	25/29 (86.2)	6/25 (24)	2/6; 1/1000 IDT 2/6; 1/100 IDT 2/6; 1/10 IDT
Infliximab	3/3 (100)	1/3 (33.3)	1/10 IDT
Trastuzumab	3/3 (100)	0	
Cetuximab	2/2 (100)	0	
Pertuzumab	2/2 (100)	1/2 (50)	1/1000 IDT
Brentuximab	2/2 (100)	1/2 (50)	1/1000 IDT
Nivolumab	1/2 (50)	0	
Tocilizumab	1/1 (100)	1/1 (100)	1/1000 IDT
Filgrastim	1/1 (100)	0	

	Median (range)
Serum tryptase level, ng/mL	
Baseline (in 37 patients)	2.42 (1–8.89)
During BTR (in 14 patients)	4.59 (1.37–24.60)
Serum IL‐6 level, pg/mL	
Baseline (in 1 patient)	10.00
During BTR (in 3 patients)	6.73 (4.26–203)

Abbreviations: BTR, breakthrough reaction; IDT, intradermal test; ST, skin test.

^a^ The patients who were evaluated for atopy.

**TABLE 2:** Type of biological drugs, indications, severity of the initial reaction, skin test results, BTR rate and drug episode that initial HSR occurred.

Biologics name indication (patient, n)	Drug episode that iHSR occurred	Grade of iHSR			IDT, *n* pos./neg.	Patients *n*,/RDD	BTR/RDD
		I	II	III			
Rituximab	1st, 2nd, 3rd, 4th, 5th					29/176	30/176
Hematologic malignancies^a^(23)		0	10	13	6/15		
Connective tissue diseases^b^(4)		0	4	0	0/3		
Autoimmune diseases^c^ (2)		0	2	0	0/1		
Infliximab	2nd, 4th, 16th					3/10	1/10
Ulcerative colitis (1)		0	1	0	0/1		
Crohn disease (1)		0	0	1	0/1		
Ankylosing spondylitis (1)		0	1	0	1/0		
Pertuzumab	3rd, 60th					2/24	2/24
Breast cancer (2)		0	1	1	1/1		
Brentuximab	3rd, 5th					2/7	1/7
Hodgkin lymphoma (2)		0	0	2	1/1		
Nivolumab	1st					2/9	1/9
Hodgkin lymphoma (1)		0	1	0	0/1		
Melanoma (1)		0	0	1			
Cetuximab	1st, 3rd					2/12	0/12
Colorectal cancer (2)		0	0	2	0/2		
Trastuzumab	1st, 5th					3/11	1/11
Breast cancer (3)		0	1	2	0/3		
Tocilizumab	12th					1/6	0/6
Rheumatoid arthritis (1)		0	0	1	1/0		
Filgrastim	1st					1/3	0/3
Breast cancer (1)		0	0	1	0/1		

Abbreviations: IDT, intradermal test; iHSR, initial hypersensitivity reaction; Neg, negative; Pos, positive; RDD, rapid drug desensitization.

^a^ Non‐Hodgkin lymphoma, Chronic lymphocytic leukemia, Waldenstrom macroglobulinemia.

^b^ Rheumatoid arthritis, systemic lupus erythematosus, Sjogren's syndrome.

^c^ Pemphigus vulgaris.

We evaluated atopy with SPT in 21 patients; three were positive for pollen and two were positive for house dust mites. The remaining 16 patients were nonatopic (Table 1).

The most common disease requiring biologicals in our study population was non‐Hodgkin's lymphoma (*n*: 17/45, 37.7%), followed by breast cancer (*n*: 6/45, 13.3%). The other indications were other malignancies, connective tissue diseases and autoimmune diseases (Table 2).

**FIGURE 1:**
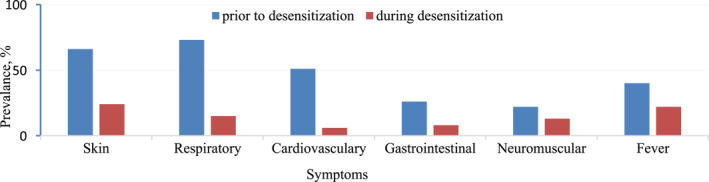
Symptom percentages during initial HSRs and during BTR (calculated accounting to the total amount of desensitization). BTR, breakthrough reactions.

In our study, rituximab was the most frequently responsible biological agent for HSRs. During the treatment with biologics, majority of the patients reacted at the first infusion (*n*: 29/45, 64.4%) and the names of the biologicals and reacted infusion numbers are shown in Table 2. The majority of initial HSRs were grade 3 (*n*: 24/45, 53.3%) or grade 2 (*n*: 21/45, 46.6%); none of them were grade 1. In patients with grade 2 or grade 3 HSR in severity, even if skin tests were negative, RDD was performed. In terms of clinical presentation, respiratory symptoms were the most common (*n*: 33/45, 73.2%), followed by skin involvement (*n*: 30/45, 66.6%; Figure 1).

Skin prick tests with the implicated biologics were performed in 40/45 (88.8%) patients but could not be performed in five patients because of the use of high‐dose systemic steroids and antihistamines on test time, limitations in timing, severe skin disease such as pemphigus vulgaris, or other non‐clinical reasons (such as patients' preference). None of the patients was positive for SPTs. Only 10/40 (25%) of the skin tested patients were positive for IDT (Table 1). Patients with grade 2 and 3 initial HSRs had positivity on IDT at rates of 27.8% and 22.7%, respectively. There was no correlation between skin test positivity and severity of initial HSRs and BTRs (*p*: 0.731, *p*: 0.923, respectively).

Initial reactions presented with three patterns as: type I (IgE or non–IgE‐mediated mast cell degranulation, n: 20/45, 44.4%), CRS (*n*: 12/45, 26.7%), and mixed reactions (*n*: 13/45, 28.8%) in our study. There was no association between the initial phenotype of HSRs and age, drug cycle, severity of HSR, tryptase levels baseline and during BTR, comorbidities, or atopy. However, there was an association between the initial phenotype of HSR and gender, SPT and IDT positivity. Most of the patients were female in Type I and mixed reaction (*p*: 0.001). A positive skin test was associated with Type I (*p*: 0.046), as expected (Table 3).

**TABLE 3:** Comparisons of risk factors for the phenotypes of initial HSRs.

Variables	Type 1, *n*: 20	CRS, *n*: 12	Mixed, *n*: 13	*p* value
Sex, *n* (%)				
Male	3 (15%)	9 (75%)	2 (15.4%)	**<0.001**
Female	17 (85%)	3 (25%)	11 (84.6%)	
Age (years)^a^	54 (20–69)	59 (23–69)	56 (30–68)	0.470
Drug cycle^a^	1 (1–60)	1 (1–16)	1 (1–3)	0.248
Baseline tryptase levels (ng/mL)^a^	1.61 (1.0–7.82)	3.45 (1.11–7.23)	2.66 (1.06–8.89)	0.182
Tryptase during BTR levels (ng/mL)^a^	2.09 (1.37–4.63)	5.31 (4.13–9.02)	5.93 (1.38–24.06)	0.139
Skin test with biologics, *n* (%)				
Negative	14/18 (77.8%)	9/9 (100%)	7/13 (53.8%)	**0.046**
Positive	4/18 (22.2%)	0/9 (0%)	6/13 (46.2%)	
Atopy, *n* (%)				
Yes	1/10 (10%)	1/3 (33.3%)	3/8 (37.5%)	0.363
No	9/10 (90%)	2/3 (66.7%)	5/8 (62.5%)	
Severity of initial HSR, *n* (%)				
Grade 2	8 (40%)	6 (50%)	7 (53.8%)	0.712
Grade 3	12 (60%)	6 (50%)	6 (46.1%)	
Allergic comorbidity, *n* (%)				
Allergic rhinitis	3 (15%)	0 (0%)	1 (7.6%)	0.282
Asthma	2 (10%)	0 (0%)	0 (0%)	
Both allergic rhinitis and asthma	0 (0%)	0 (0%)	1 (7.6%)	
None	15 (75%)	12 (50%)	11 (84.6%)	
Another comorbidity, *n* (%)^b^				
Yes	13 (65%)	7 (58.3%)	5 (38.5%)	0.317
No	7 (35%)	5 (41.7%)	8 (61.5%)	

*Note*: The bold values point out statistically significance.

Abbreviations: BTR, breakthrough reactions; HSR, hypersensitivity reactions.

^a^ Median, range.

^b^ Endocrinological, cardiovascular and neurological comorbidities.

BTRs developed in 36 infusions of 258 RDDs with a severity were grade 1: 11 of 36 (30.5%); grade 2: 21 of 36 (58.3%); and grade 3: 4 of 36 (11.1%). 19 of 36 (52.7%) BTRs occurred during the first RDD. Twenty‐three of 45 patients tolerated RDD perfectly without reactions and additional three patients developed BTR after initial RDD leading a total of (57.8%) 26/45 of patients did not develop BTR during initial RDD. A total of 22 patients (48.8%) developed BTR during any RDD, but 86.3% (*n*: 19/22) of these patients developed BTR during initial RDD. Due to clinical necessity, each patient underwent different numbers of desensitizations, and BTR did not develop in each desensitization; moreover, whether or not BTR developed, its grade and phenotype varied even for the same patient (Table 2). To compare severity (grade) and phenotype of the BTR compared with the initial ones, as it has been done in the study of Isabwe et al., we compared severity (grade) and phenotype of BTRs developed during the first desensitization to severity (grade) and phenotype of initial HSR to evaluate differences between these two groups.^5^ Comparing the severity and phenotype between HSR and BTR during first desensitization, Grade 2 and Grade 3 BTRs were significantly decreased (*p*: 0.035, *p*: 0.001, respectively) during RDD. Additionally, there was a significant decrease in phenotypes of type 1 and mixed type reactions during first desensitization (*p*: 0.004, *p*: 0.002, respectively). There was a decrease in the CRS phenotype, but it was not statistically significant (Table 4).

**TABLE 4:** Severity and pheonotype comparison between iHSR and BTR during first desensitization.


Variables	iHSR (*n*: 45)	BTR during first desensitization (*n*: 19)	*p* value
Severity			
Grade 1	0 (0%)	4 (21%)	0.125
Grade 2	21 (46.7%)	12 (63.1%)	**0.035**
Grade 3	24 (53.3%)	3 (15.7%)	**0.001**
Phenotype			
Type 1	20 (44.4%)	8 (42.1%)	**0.004**
CRS	12 (26.7%)	8 (42.1%)	0.344
Mixed	13 (28.9%)	3 (15.7%)	**0.002**

*Note*: The bold values point out statistically significance.

Abbreviations: BTR, breakthrough reactions; CRS, cytokine‐release syndrome; iHSR, initial hypersensitivity reactions; ND, not done.

Twenty two patients (48.8%) developed a total of 36 BTRs. Among patients with BTRs, 63.6% (14/22) had only one, 18.1% (4/22) had two, 13.6% (3/22) had three, and only 4.5% (1/22) had five BTRs during the RDDs. In general, most of the reactions during RDDs were milder than initial reactions. BTRs were most likely to occur at the 12th step of the first desensitization. The median number of RDDs was five (range: 1–20). Four RDDs could not be completed in four patients with rituximab because of anaphylactic shock and fever that was unresponsive to treatment (also one patient had severe pemphigus and ventricular extrasystoles during BTR). One patient's RDD was stopped by the Hematology Department's decision. For one patient, we divided the total therapeutic dose of rituximab into 1/3 and 2/3, administered these divided doses as desensitization on separate days because of the severity comorbidities of heart and kidney failure and the desensitization was completed successfully. A total of 258 RDDs was performed in 45 patients and 98.4% of this desensitization were completed successfully.

Twenty one of 36 BTRs (58.3%) were type I reaction, 12 (33.3%) were CRS and three (8.3%) were mixed reactions, respectively. There was no significant association between the risk of BTRs and age, drug cycle, SPT positivity/dilutions of IDT, gender, comorbidities, or atopy. While BTR occurred in 15/21 (71.4%) of patients with HSR grade 2 in severity, that occurred in 7/24 (29.2%) of patients with HSR grade 3 in severity (*p*:0.005; Table 5). Thirty‐seven patients had recorded baseline tryptase levels median: 2.42 mg/L (range: 1–8.89), and among them tryptase levels were measured both at baseline and during BTRs in 14 patients. A significant elevation in tryptase levels (1.2 times baseline + 2 mg/L) was found in two patients during BTRs with a median tryptase level of 4.59 mg/dl (range: 1.37–24.60). The tryptase level of the patient was over 11.5 mg/dl during BTR and the patient had Grade 1 BTR.

**TABLE 5:** Comparison of risk factors between RDD with and without BTRs.

Variables	RDD without BTRs (*n*: 23)	RDD with BTRs (*n*: 22)	*p*‐value
Sex, *n* (%)			
Male	7 (30.4%)	7 (31.8%)	0.920
Female	16 (69.6%)	15 (68.2%)	
Age, (years)^a^	55 (23–69)	57.5 (20–69)	0.304
Drug cycle^a^	1 (1–60)	1 (1–4)	0.290
Baseline tryptase levels (ng/mL)^a^	1.8 (1–7.8)	3.7 (1–8.9)	0.061
Tryptase level during BTR (ng/mL)^a^	No BTR	4.59 (1.37–24.60)	‐
Skin test with biologics, *n* (%)		1	
Negative	16/21 (76.2%)	4/19 (73.7%)	1.000
Positive	5/21 (23.8%)	5/19 (26.3%)	
Atopy, *n* (%)			
Yes	2/11 (18.2%)	3/10 (30.0%)	0.635
No	9/11 (81.8%)	7/10 (70.0%)	
The phenotypes of HSR, *n* (%)			
Type 1	13 (56.5%)	7 (31.8%)	0.157
CRS	6 (26.1%)	6 (27.3%)	
Mixed	4 (17.4%)	9 (40.9%)	
Severity of initial HSR, *n* (%)			**0.005**
Grade II	6 (26.1%)	15 (68.2%)	
Grade III	17 (73.9%)	7 (31.8%)	
Allergic comorbidity, *n* (%)			
Allergic rhinitis	2 (8.7%)	2 (9.1%)	0.781
Asthma	1 (4.3%)	1 (4.5%)	
Both allergic rhinitis and asthma	0 (0.0%)	1 (4.5%)	
None	20 (87.0%)	18 (81.8%)	
Another comorbidity^b^			
Yes	11 (47.8%)	14 (63.6%)	0.286
No	12 (52.2%)	8 (36.4%)	

*Note*: The bold values point out statistically significance.

Abbreviations: BTR, breakthrough reactions; HSR, hypersensitivity reactions; RDD, rapid drug desensitization.

^a^ Median (range).

^b^ Endocrinological, cardiovascular and neurological comorbidities.

## 4 DISCUSSION

We presented our experience regarding 258 RDDs to nine biological agents in 45 patients with immediate HSRs to biologics. One of the large series of 526 RDDs with 16 biologics was performed in 104 patients by Isabwe et al.^5^ Another study from the same department, 105 RDDs with three biologics were performed in 23 patients by Brennan et al.^27^ As in our study, the most common indications for treatment were hematologic malignancies in these two cohorts.

All biologics can cause HSRs, which may occur rapidly and can be severe. Rituximab was the most common drug involved in HSRs in our series that is similar to previous reports. In terms of clinical presentations, the most frequently observed type of reaction reported was cutaneous, followed by cardiovascular, respiratory, and throat tightness.^5^, ^12^, ^27^ In our study, respiratory symptoms were the most frequent manifestation followed by cutaneous symptoms. This may be related to the patient selection for RDD since our study population had grade II and grade III reactions in severity. HSRs to biologics can occur during the first exposure (i.e., cetuximab, trastuzumab, and rituximab) or after multiple exposures (i.e., rituximab and infliximab).^23^, ^27^, ^28^, ^29^

In rituximab‐focused studies, the majority of patients reacted to the drug at the first exposure.^27^, ^29^, ^30^, ^31^ In contrast, Brennan et al. recorded in their series that for infliximab and trastuzumab, the majority of HSRs were observed after multiple exposures.^27^ In keeping with this, we observed that most patients reacted to the rituximab at the first exposure but for infliximab, pertuzumab, brentuximab, and tocilizumab, HSRs were observed after multiple exposures. It is unexpected that IgE mediated reactions to rituximab occur on first exposure however sensitization to cross‐reactive antigens might also bring about preformed IgE production in the absence of drug exposure.^32^, ^33^, ^34^, ^35^, ^36^ Skin tests, either SPTs or IDTs, to the culprit biological agents can be performed in patients with immediate HSRs to assess the presence of specific IgE antibodies to the implicated agent.^27^, ^28^, ^29^ One of the main limitations of skin testing for biologicals is the lack of standardized procedures including non‐irritant drug concentrations.^21^ The other limitation is the high cost of biologicals. In the largest series, skin testing could be performed for 10 biologics in 58 of 104 (55.7%) patients and positivity was obtained in 24 patients (41%, 9 with SPT, 15 with IDT) and skin tests were found negative in 34 patients (59%).^5^ In a previous study from the same group, skin testing was performed for 3 biologics in 18 of 23 patients (78.2%) with 13 (72%) positive and 5 (27%) negative results.^27^ Sloane et al. recorded in their series that skin testing was performed for 6 biologics in 31 of 41 (75.6%) patients with 20 positive (64.5%) and 11 negative (35.4%) results.^37^ In our study, skin testing was performed in 40 of 45 patients (88.8%) except five patients. None of the patients had positive SPTs but IDT, positivity was observed in six of 25 patients with rituximab, 1 of 3 patients with infliximab, 1 of 2 patients with pertuzumab, 1 of 2 patients with brentuximab and 1 of 1 patient with tocilizumab. This condition may be related to the biologics used for skin tests. Compared to all these series, we performed skin tests with implicated biological agents in a very high percentage of cases (88.8%). In our study, 4/10 (40%) of the subjects with positive skin test were type 1 phenotype and 6/10 (60%) were mixed reaction phenotype during HSRs.

In our experience, 98.4% of 258 RDDs to biologics were completed successfully, with a rate of BTRs by (36/258 infusions, 13.9%) of all RDDs. Most of the BTRs were milder than the initial reactions and 19 of 36 (52.7%) BTRs occurred during the first RDD. In the largest series of desensitizations, BTRs occurred as 15% mild (grade I), 8% moderate (grade II), and 0.4% severe reactions (grade III).^5^

Data reported about risk factors in patients with BTRs in biologics are limited. In our study, there was no significant association between the risk of BTRs and age, drug cycle, SPT or IDT positivity, gender, comorbidities, or atopy. While BTR occurred in 15/22 (68.2%) of patients with HSR grade 2 in severity, it occurred in 7/22 (31.8%) of patients with HSR grade 3 in severity (*p*:0.005; Table 5). Although we did not systematically evaluate this situation, we may speculate that it may be related to the intensity of premedication since we added aspirin and/or leukotriene antagonist and/or fluid support to more severe cases with initial HSR.

Some trials find a correlation between positive skin test results and greater severity of HSRs.^5^ Compared with the trial, neither this study^38^ nor our study reported an association between skin test positivity and severity of HSRs and BTRs (*p*: 0.731, *p*: 0.923, respectively). More trials are needed on this issue. Desensitization with rituximab has been defined in some case reports or case series,^16^, ^29^, ^30^, ^31^, ^39^, ^40^ but in our study we performed 176 RDDs in total with rituximab in 29 patients, and this appears to be the highest number for rituximab desensitization reported to date outside the USA. Our BTR rate was 17% for rituximab RDD, lower than that reported by Sloane et al. (28%).^37^

A review of case reports on HSRs to cetuximab showed that many HSRs occurred within minutes of patients' first exposure to the drug.^2^, ^11^, ^40^ In most patients, IgE antibodies specific for galactose‐α‐1,3‐galactose, possibly generated by tick exposure, are present in the serum before therapy with cetuximab.^41^ We had two patients with a grade III reaction and negative skin tests to cetuximab after the first and third exposures, respectively, and they received 12 successful RDDs. However, these patients were not aware as to whether they had been exposed to tick bites. Infliximab is an effective monoclonal antibody against tumor necrosis factor.^23^, ^42^ In our trial, three patients completed 10 RDDs with infliximab. Trastuzumab is indicated for the treatment of breast cancer or metastatic gastrointestinal adenocarcinoma. As a humanized mouse monoclonal antibody, it has a potentially low immunogenic profile, but few HSRs have been reported.^23^, ^27^, ^42^ We had three patients with metastatic breast cancer, and skin testing was negative for them.

In the studies with RDD to biological agents, cutaneous reactions were the most prevalent presentations during desensitization procedure as in our study.^5^, ^27^, ^28^ Our BTR rate was 13.9%, lower than that reported by Brennan et al. (29%).^27^ The majority of our desensitized subjects were already on steroids as part of their treatment protocols. Furthermore, for patients with cutaneous and respiratory reactions during the initial reaction or desensitization, we adjusted the protocol by adding aspirin and montelukast, respectively. We believe that all these interventions could have reduced the BTRs in our study. Consequently, we were able to complete the entire RDD protocol in all patients, except in four desensitization procedures with fever unresponsive to treatment, ventricular extrasystole and patients' preference.

Our study has disadvantages and advantages. The retrospective nature of the study was a disadvantageous. However, considering the importance of this procedure, we have been systematically collecting details from such cases since our first case with RDD. Therefore, we are confident that we had all the relevant data. The other disadvantage was that most cases were desensitized with one biologics, rituximab (29 subjects with 176 desensitization). This was because the study group mainly consisted of hematologic malignancies, and rituximab has been prescribed as the most efficient treatment option for such cases. On the other hand, such a high number of RDDs with rituximab was an advantage because, to the best of our knowledge, this means we had the highest number of reported RDDs with rituximab to date outside the USA. As another advantage, we performed a high number of RDDs along with skin testing with implicated biological agents in a very high percentage of cases. The other advantage of our study was that we performed skin test and desensitization with nivolumab and filgrastim. In addition, the total number of RDDs with nivolumab was higher than in other studies and cases in the literature.

In conclusion, RDD in patients with immediate‐type HSRs to biological agents provides a safe and effective management strategy, and it may be an option for patients who react to first‐line treatment and do not have a suitable alternative.

## AUTHOR CONTRIBUTIONS

**Döne Gülçin Unutmaz Erkaya**: Conceptualization; investigation; writing—original draft; writing—review & editing; methodology; validation; visualization; formal analysis; software; data curation; resources; supervision. **Makbule Seda Bayrak Durmaz**: Data curation; conceptualization; investigation; methodology. **Begüm Görgülü Akın**: Data curation; conceptualization; methodology. **Sevim Bavbek**: Investigation; conceptualization; methodology; validation; visualization; writing—review & editing; formal analysis; project administration; data curation; supervision; resources.

## CONFLICT OF INTEREST STATEMENT

The authors declare that the research was conducted in the absence of any commercial or financial relationships that could be construed as a potential conflict of interest.

## Supporting information

Table S1

## Data Availability

The data that support the findings of this study are available from the corresponding author upon reasonable request.

## References

[1] Cheifetz A , Smedley M , Martin S , et al. The incidence and management of infusion reactions to infliximab: a large center experience. Am J Gastroenterol. 2003;98(6):1315‐1324. 10.1111/j.1572-0241.2003.07457.x 12818276

[2] Chung KY , Shia J , Kemeny NE , et al. Cetuximab shows activity in colorectal cancer patients with tumors that do not express the epidermal growth factor receptor by immunohistochemistry. J Clin Oncol. 2005;23(9):1803‐1810. 10.1200/JCO.2005.08.037 15677699

[3] Cook‐Bruns N . Retrospective analysis of the safety of Herceptin® immunotherapy in metastatic breast cancer. Oncology. 2001;61(2):58‐66. 10.1159/000055403 11694789

[4] Makris MP , Papadavid E , Zuberbier T . The use of biologicals in cutaneous allergies–present and future. Curr Opin Allergy Clin Immunol. 2014;14(5):409‐416. 10.1097/ACI.0000000000000096 25102106

[5] Isabwe GAC , Neuer MG , de las Vecillas Sanchez L , Lynch D.‐M , Marquis K , Castells M . Hypersensitivity reactions to therapeutic monoclonal antibodies: phenotypes and endotypes. J Allergy Clin Immunol. 2018;142(1):159‐170. 10.1016/j.jaci.2018.02.018 29518427

[6] Grillo‐López AJ , White CA , Varns C , et al. Overview of the clinical development of rituximab: first monoclonal antibody approved for the treatment of lymphoma. Semin Oncol. 1999:66‐73. 10561020

[7] Baert F , Noman M , Vermeire S , et al. Influence of immunogenicity on the long‐term efficacy of infliximab in Crohn's disease. N Engl J Med. 2003;348(7):601‐608. 10.1056/NEJMoa02088 12584368

[8] Maini R , St Clair EW , Breedveld F , et al. Infliximab (chimeric anti‐tumour necrosis factor α monoclonal antibody) versus placebo in rheumatoid arthritis patients receiving concomitant methotrexate: a randomised phase III trial. Lancet. 1999;354(9194):1932‐1939. 10.1016/s0140-6736(99)05246-0 10622295

[9] Soykan I , Ertan C , Özden A . Severe anaphylactic reaction to infliximab: report of a case. Am J Gastroenterol. 2000;95(9):2395‐2396. 10.1111/j.1572-0241.2000.02349.x 11007258

[10] Herceptin prescribing information . Genentech; revised 3/2009. Available at:. Accessed July 1. http://www.gene.com/gene/products/information/pdf/herceptin‐prescribing.pdf

[11] O'Neil BH , Allen R , Spigel DR , et al. High incidence of cetuximab‐related infusion reactions in Tennessee and North Carolina and the association with atopic history. J Clin Oncol. 2007;25(24):3644‐3648. 10.1200/JCO.2007.11.7812 17704414

[12] Castells M , Bonamichi‐Santos R . Drug hypersensitivity. In: Clinical Immunology. Elsevier; 2019:649‐667.

[13] del Carmen Sancho M , Breslow R , Sloane D , Castells M . Desensitization for hypersensitivity reactions to medications. In: Adverse Cutaneous Drug Eruptions. Karger Publishers; 2012:217‐233. 10.1159/000335637 22613865

[14] Kendirlinan R , Gümüşburun R , Çerçi P , et al. Rapid drug desensitization with chemotherapeutics (platins, taxanes, and others): a single‐center retrospective study. Int Arch Allergy Immunol. 2019;179(2):114‐122. 10.1159/000496745 30893688

[15] Bavbek S , Kendirlinan R , Çerçi P , et al. Rapid drug desensitization with biologics: a single‐center experience with four biologics. Int Arch Allergy Immunol. 2017;171(3‐4):227‐233. 10.1159/000454808 28049204

[16] Gorgulu B , Seval G , Kendirlinan R , Toprak S , Ozcan M , Bavbek S . Rapid drug desensitization with rituximab in 24 cases: a single‐center experience. J Investig Allergol Clin Immunol. 2019;29(6):468‐470. 10.18176/jiaci.0445 31530515

[17] Brown SG . Clinical features and severity grading of anaphylaxis. Allergy Clin Immunol. 2004;114(2):371‐376. 10.1016/j.jaci.2004.04.029 15316518

[18] Fouda GE , Bavbek S . Rituximab hypersensitivity: from clinical presentation to management. Front Pharmacol. 2020;11:572863. 10.3389/fphar.2020.572863 33013416 PMC7508176

[19] Bavbek S , Pagani M , Alvarez‐Cuesta E , et al. Hypersensitivity reactions to biologicals: an EAACI position paper. Allergy. 2022;77(1):39‐54. 10.1111/all.14984 34157134

[20] Valent P , Akin C , Arock M , et al. Definitions, criteria and global classification of mast cell disorders with special reference to mast cell activation syndromes: a consensus proposal. Int Arch Allergy Immunol. 2012;157(3):215‐225. 10.1159/000328760 22041891 PMC3224511

[21] Brockow K , Garvey L , Aberer W , et al. Skin test concentrations for systemically administered drugs–an ENDA/EAACI Drug Allergy Interest Group position paper. Allergy. 2013;68(6):702‐712. 10.1111/all.12142 23617635

[22] Caiado J , Brás R , Paulino M , Costa L , Castells M . Rapid desensitization to antineoplastic drugs in an outpatient immunoallergology clinic: outcomes and risk factors. Ann Allergy Asthma Immunol. 2020;125(3):325‐333. 10.1016/j.anai.2020.04.017 32353405

[23] Castells MC , Tennant NM , Sloane DE , et al. Hypersensitivity reactions to chemotherapy: outcomes and safety of rapid desensitization in 413 cases. J Allergy Clin Immunol. 2008;122(3):574‐580. 10.1016/j.jaci.2008.02.044 18502492

[24] Morales AR , Shah N , Castells M . Antigen‐IgE desensitization in signal transducer and activator of transcription 6‐deficient mast cells by suboptimal doses of antigen. Ann Allergy Asthma Immunol. 2005;94(5):575‐580. 10.1016/s1081-1206(10)61136-2 15945561

[25] Sancho‐Serra MdelCSM , Castells M . Rapid IgE desensitization is antigen specific and impairs early and late mast cell responses targeting FcεRI internalization. Eur J Immunol. 2011;41(4):1004‐1013. 10.1002/eji.201040810 21360700

[26] Breslow RG , Caiado J , Castells MC . Acetylsalicylic acid and montelukast block mast cell mediator–related symptoms during rapid desensitization. Ann Allergy Asthma Immunol. 2009;102(2):155‐160. 10.1016/S1081-1206(10)60247-5 19230468

[27] Brennan PJ , Bouza TR , Hsu FI , Sloane DE , Castells MC . Hypersensitivity reactions to mAbs: 105 desensitizations in 23 patients, from evaluation to treatment. J Allergy Clin Immunol. 2009;124(6):1259‐1266. 10.1016/j.jaci.2009.09.009 19910036

[28] Hong DI , Bankova L , Cahill KN , Kyin T , Castells MC . Allergy to monoclonal antibodies: cutting‐edge desensitization methods for cutting‐edge therapies. Expet Rev Clin Immunol. 2012;8(1):43‐54. 10.1586/eci.11.75 22149339

[29] Ataca P , Atilla E , Kendir R , Bavbek S , Ozcan M . Successful desensitization of a patient with rituximab hypersensitivity. Case Reports Immunol. 2015;2015(1):1‐4. 10.1155/2015/524507 PMC4320878 25685566

[30] Abadoglu O , Epozturk K , Atayik E , Kaptanoglu E . Successful rapid rituximab desensitization for hypersensitivity reactions to monoclonal antibodies in a patient with rheumatoid arthritis: a remarkable option. J Investig Allergol Clin Immunol. 2011;21(4):319‐321. 21721382

[31] Lebel E , Ben‐Yehuda D , Bohbot E , Dranitzki Z , Shalit M , Tal Y . Hypersensitivity reactions to rituximab: 53 successful desensitizations in 7 patients with severe, near‐fatal reactions. J Allergy Clin Immunol Pract. 2016;4(5):1000‐1002. 10.1016/j.jaip.2016.05.013 27329468

[32] Bush RK , Wood RA , Eggleston PA . Laboratory animal allergy. J Allergy Clin Immunol. 1998;102(1):99‐112. 10.1016/s0091-6749(98)70060-0 9679853

[33] Matsui EC , Simons E , Rand C , et al. Airborne mouse allergen in the homes of inner‐city children with asthma. J Allergy Clin Immunol. 2005;115(2):358‐363. 10.1016/j.jaci.2004.11.007 15696095

[34] Matsui EC , Wood RA , Rand C , Kanchanaraksa S , Swartz L , Eggleston PA . Mouse allergen exposure and mouse skin test sensitivity in suburban, middle‐class children with asthma. J Allergy Clin Immunol. 2004;113(5):910‐915. 10.1016/j.jaci.2004.02.034 15131574

[35] Park A , Edwards M , Donaldson M , Ghatei M , Meeran K . Interfering antibodies affecting immunoassays in woman with pet rabbits. BMJ. 2003;326(7388):541‐542. 10.1136/bmj.326.7388.541 12623918 PMC1125424

[36] Platts‐Mills TA , Satinover SM , Naccara L , et al. Prevalence and titer of IgE antibodies to mouse allergens. J Allergy Clin Immunol. 2007;120(5):1058‐1064. 10.1016/j.jaci.2007.06.032 17767949

[37] Sloane D , Govindarajulu U , Harrow‐Mortelliti J , et al. Safety, costs, and efficacy of rapid drug desensitizations to chemotherapy and monoclonal antibodies. J Allergy Clin Immunol Pract. 2016;4(3):497‐504. 10.1016/j.jaip.2015.12.019 26895621

[38] Wong JT , Long A . Rituximab hypersensitivity: evaluation, desensitization, and potential mechanisms. J Allergy Clin Immunol Pract. 2017;5(6):1564‐1571. 10.1016/j.jaip.2017.08.004 29122155

[39] Vultaggio A , Matucci A , Nencini F , et al. Drug‐specific Th2 cells and IgE antibodies in a patient with anaphylaxis to rituximab. Int Arch Allergy Immunol. 2012;159(3):321‐326. 10.1159/000336839 22846615

[40] Amorós‐Reboredo P , Sánchez‐López J , Bastida‐Fernández C , et al. Desensitization to rituximab in a multidisciplinary setting. Int J Clin Pharm. 2015;37(5):744‐748. 10.1007/s11096-015-0136-x 25999014

[41] Chung CH , Mirakhur B , Chan E , et al. Cetuximab‐induced anaphylaxis and IgE specific for galactose‐α‐1, 3‐galactose. N Engl J Med. 2008;358(11):1109‐1117. 10.1056/NEJMoa074943 18337601 PMC2361129

[42] Galvão VR , Castells MC . Hypersensitivity to biological agents—updated diagnosis, management, and treatment. J Allergy Clin Immunol Pract. 2015;3(2):175‐185. 10.1016/j.jaip.2014.12.006 25754718

