# Supplementary material for: Retrospective analysis of rapid drug desensitization with biologic agents: A single center experience
Source: Clin Transl Allergy. 2024 Oct 21;14(10):e12397. doi: 10.1002/clt2.12397 (PMC11493553; doi:10.1002/clt2.12397)
Supplement: Supplementary file 1 — Table S1 [file CLT2-14-e12397-s001.docx]

| **Supplementary Table 1**: The standard 12- step, 3- bag desensitization protocol from the Brigham and Women’s Hospital Rapid Drug Desensitization Program^23^ | | | | |
| --- | --- | --- | --- | --- |
| **Step** | **Solution** | **Rate (ml/h)** | **Time (min)** | **Volume infused per step (ml)** |
| 1 | A | 2.5 | 15 | 0.625 |
| 2 |  | 5.0 | 15 | 1.25 |
| 3 |  | 10.0 | 15 | 2.50 |
| 4 |  | 20.0 | 15 | 5.00 |
| 5 | B | 5.0 | 15 | 1.25 |
| 6 |  | 10.0 | 15 | 2.50 |
| 7 |  | 20.0 | 15 | 5.00 |
| 8 |  | 40.0 | 15 | 10.0 |
| 9 | C | 10.0 | 15 | 2.50 |
| 10 |  | 20.0 | 15 | 5.00 |
| 11 |  | 40.0 | 15 | 10.0 |
| 12 |  | 80.0 | 175 | 232.5 |
| *Solution A: 250 ml of 1:100 concentration;*  *Solution B: 250 ml of 1:10 concentration;*  *Solution C: 250 ml of 1:1 concentration* | | | | |
